# Enhanced Coercivity and Tb Distribution Optimization of Sintered Nd-Fe-B Magnets by TbF_3_ Grain Boundary Diffusion Facilitated by Ga

**DOI:** 10.3390/molecules30030594

**Published:** 2025-01-28

**Authors:** Ling Wang, Wenjiao Li, Xiaopeng Wang, Zejun Deng, Shujuan Gao

**Affiliations:** 1 Department of Chemical and Material Engineering, Lyuliang University, Lvliang 033001, China; 20151031@llu.edu.cn (W.L.); wangxpfoxwish@163.com (X.W.); 2 School of Materials Science and Engineering, Central South University, Changsha 410083, China; zejun.deng@csu.edu.cn

**Keywords:** Nd-Fe-B, grain boundary diffusion, electrophoretic deposition, magnetic properties

## Abstract

The grain boundary diffusion process employing a mixed diffusion source, comprising heavy rare-earth elements and low-melting metals, significantly enhances the coercivity (H_cj_) of sintered Nd-Fe-B magnets. In the present study, Tb and Ga were deposited onto the surface of Nd-Fe-B magnets to serve as a diffusion source for improving hard magnetic properties. The effects of varying deposition sequences of Tb and Ga on the magnetic properties and microstructure of the magnets were analyzed. The findings demonstrate that TbF_3_ grain boundary diffusion facilitated by Ga effectively increases the efficiency of Tb substitution, leading to enhanced coercivity. When Tb and Ga are deposited simultaneously, coercivity shows a notable improvement of 53.15% compared to the untreated magnet, with no reduction in remanence. Additionally, thermal stability is enhanced, resulting in superior overall magnetic properties. Microstructural analysis reveals that Ga promotes the diffusion of Tb into the magnet. In the magnet where Tb and Ga are co-deposited, the formation of a thinner and more uniform (Nd,Tb)_2_Fe_14_B shell–core structure, along with the greater infiltration depth of Tb, leads to a broader distribution of core–shell structures within the magnet. This effectively increases the anisotropy fields (*H*_A_) of the main phase grains, preventing the nucleation of antiferromagnetic domains at the edges of main-phase grains, thereby enhancing coercivity. Furthermore, the corrosion resistance of the magnet subjected to mixed diffusion is improved. This study provides a foundation for producing highly efficient magnets with a lower content of heavy rare-earth elements. The simplicity and flexibility of the process make it highly suitable for industrial applications.

## 1. Introduction

Permanent magnet materials are crucial in the realms of modern science, technology, and industry. From everyday items like smartphones, headphones, and hard disk drives, to core components in new energy vehicles, 5G communications, high-speed rail transportation, and advanced manufacturing equipment like MRI machines and precision instruments, permanent magnet materials are indispensable. These applications not only rely on the high energy products and stable magnetic performance of permanent magnetic materials, but also on their lightweight, high efficiency, and environmentally friendly and energy-saving characteristics. Among the various permanent magnet materials, Nd-Fe-B stands out due to its exceptionally high magnetic energy products and coercivity, making it the ideal choice for achieving high performance, miniaturization, and lightweight in electronic and electromechanical products.

As the development of electric vehicles and robotics technology continues, the status of Nd-Fe-B permanent magnets will become increasingly prominent. However, their low Curie temperature and poor stability at elevated temperatures restrict their applicability [1,2]. To enhance high-temperature coercivity, heavy rare earth (HRE) elements that can form a (HRE, Nd)_2_Fe_14_B phase with higher magnetic anisotropy fields, such as Dy or Tb, are commonly introduced. However, the addition of Dy or Tb decreases the maximum energy product ((*BH*)*_max_*) and remanence (B_r_) due to the antiferromagnetic coupling between Dy or Tb atoms and Fe atoms within the (Nd, HRE)_2_Fe_14_B lattice, leading to a reduction in saturation magnetization (*M_s_*) [3,4]. Furthermore, given the limited availability of heavy rare earths, reliance solely on Dy or Tb for improving magnet performance has become increasingly impractical. Research has also demonstrated [5,6,7] that antiferromagnetic domains often nucleate near grain boundaries—specifically, in the peripheral regions of the main phase grains—before expanding and growing. Consequently, confining heavy rare earth elements to the outer regions of the main phase grains can achieve enhanced coercivity while minimizing the loss of remanence caused by HRE addition. This realization led to the development of the grain boundary diffusion process (GBDP) [8,9]. During this process, heavy rare earth elements such as Dy or Tb infiltrate the magnets predominantly through grain boundaries, forming an HRE-enriched shell with higher anisotropic fields and continuous grain boundary phases that improve magnetic isolation between main phases. This method has successfully reduced heavy rare earth usage, achieving both resource conservation and cost efficiency.

GBD technology has been extensively utilized for producing magnets with low heavy rare earth (HRE) content and high coercivity [10,11,12,13]. Among the critical processes in the GBD technique, coating preparation plays a pivotal role, directly affecting the productivity, coating quality, production costs, and ultimately, the diffusion efficiency of HRE elements into the magnet, influencing the cost-effectiveness of the final GBD product. Various methods have been employed for coating magnets, including dip coating, chemical bath deposition, chemical vapor deposition, vacuum evaporation plating, and sputtering of alloys or pure metals [14,15,16,17]. However, these techniques face challenges in large-scale industrial application, such as complex procedures, high costs, uneven coating, or poor interfacial adhesion. Recently, electrophoretic deposition (EPD) technology has garnered significant attention [18,19,20,21]. This method involves depositing DyF_3_ or TbF_3_ powders dispersed in a medium onto the magnet surface under an external electric field. Compared to other techniques, EPD offers advantages such as high deposition efficiency, uniform and dense film formation, lower deposition costs, controllable coating thickness, and simpler operations, making it more suitable for industrial-scale production.

Heavy rare earth elements, such as Dy, Tb, and their corresponding oxides, fluorides, and hydrides, are frequently employed as diffusion sources. In 2012, Soderznik et al. [22] first applied DyF_3_ powder onto the surface of sintered magnets using EPD, resulting in a significant improvement in H_cj_ from 1200 to 1620 kA/m. In 2015, Cao et al. [23] used the EPD technique to deposit DyF_3_ powder on the magnet surface, leading to a 50% increase in H_cj_ from 1274 to 1911 kA/m. In 2016, Cao et al. [24] continued their work by preparing TbF_3_ diffusion magnets with varying RE content via the EPD method, suggesting that a higher RE content enhanced the Tb diffusion process. In 2023, Wang et al. [25] established that increasing the Tb content significantly boosted the coercivity of the magnet during the EPD process of TbF_3_ powder. Nevertheless, substantial increases in the usage of HRE can result in considerable cost increases. Several recent studies have investigated the use of mixed diffusion sources, combining HRE with low melting point metals (such as Zn, Cu, Al) [26,27,28,29,30]. When compared with HRE diffusion magnets, mixed diffusion magnets show a more pronounced enhancement in H_cj_. Moreover, the use of this mixed diffusion source further improves the utilization efficiency of HRE, while also lowering production costs.

Recent studies have demonstrated that adding small amounts of Ga can significantly enhance the magnetic properties of Nd-Fe-B sintered magnets [31,32]. The observed increase in coercivity has been associated with improved wettability of the liquid phase during sintering and the formation of additional Nd-rich phases at the grain boundaries. Current research on non-rare earth Ga mainly focuses on traditional doping methods, with limited reports on its use as a diffusion source for grain boundary diffusion.

In this study, TbF_3_ and Ga(NO_3_)_3_ powders were deposited onto the surface of sintered Nd-Fe-B magnets via EPD to create a diffusion source. The GBDP was then completed through heat treatment. The impact of Ga-assisted Tb grain boundary diffusion on the microstructure, magnetic properties, and corrosion resistance of sintered Nd-Fe-B magnets was evaluated.

## 2. Results and Discussion

**Figure 1:**
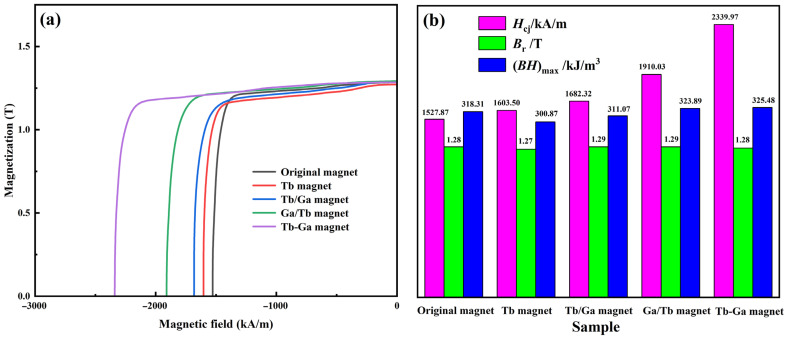
(**a**) Demagnetization curves of the original magnet, Tb magnet, Tb/Ga magnet, Ga/Tb magnet and Tb-Ga magnet at 298 K. (**b**) The histogram of the magnetic properties.

Figure 1a presents the demagnetization curves of both the original and diffused magnets at 298 K. As shown in Figure 1b, the coercivity (H_cj_) of the original sample is 1527.87 kA/m, while the remanence (B_r_) is 1.28 T. After the diffusion of Tb at the grain boundary, the coercivity of the “Tb magnet” increased to 1603.50 kA/m, representing a 4.95% improvement, while the remanence slightly decreased to 1.27 T. These findings suggest that Tb grain boundary diffusion effectively enhances coercivity, although a minor reduction in remanence occurs. It is known that Tb predominantly concentrates around the primary phase of the magnet following GBDP, aiding in the formation of the Tb_2_Fe_14_B phase. Given that the anisotropy field of this phase is considerably higher than that of the Nd_2_Fe_14_B phase and is positioned within the structure where demagnetization initiates [33], the demagnetization process is significantly reduced. In contrast to the “Tb magnet”, the coercivity of the Tb and Ga mixed diffusion magnet was substantially increased, whether the deposition occurred layer by layer or simultaneously. The coercivity of the “Tb/Ga magnet”, “Ga/Tb magnet”, and “Tb-Ga magnet” reached 1682.32 kA/m (an increase of 10.11%), 1910.03 kA/m (25.01%), and 2339.97 kA/m (53.15%), respectively. As shown in prior studies [31,32,34], Ga elements lower the melting point of the Nd-Fe alloy, enhance the wettability of the grain boundary phase, and create effective diffusion paths. This results in improved GBDP efficiency, leading to an increase in the intrinsic coercivity of sintered magnets. It is worth noting that in the case of the “Tb/Ga magnet”, after Tb deposition, the Tb film tends to peel off considerably in Ga(NO_3_)_3_ solution during the subsequent deposition stage, which causes a significant reduction in the Tb content in the diffusion source and, as a result, a less pronounced increase in coercivity.

The H_cj_ of the “Tb-Ga magnet” is the highest among the four, with an increase of up to 53.15%. This improvement is significantly greater than that observed in magnets diffused solely with Tb or those with layer-by-layer diffusion of Ga and Tb. This suggests that the simultaneous deposition of Tb and Ga provides a more substantial enhancement of coercivity.

In addition, unlike the “Tb magnet”, the magnetic remanence (B_r_) of the three magnets with mixed diffusion sources remained unchanged or slightly increased. Both the “Tb/Ga magnet” and “Ga/Tb magnet” exhibited minor enhancements in B_r_. This implies that incorporating a low-melting-point element assisted in the diffusion of Tb, resulting in a marked improvement in coercivity while keeping B_r_ nearly constant. Consequently, this led to a noticeable increase in (BH)*_max_*. Overall, introducing Ga into the diffusion source significantly improves the overall magnetic properties of the magnet and optimizes Tb utilization.

The temperature stability of a magnet indicates its capacity to preserve its magnetic properties under different external temperatures. The remanence temperature coefficient (*α*) and coercivity temperature coefficient (*β*) are commonly used to describe the temperature stability of magnets. They are defined as follows:

(1) $$\alpha =\frac{B_{r}(T)-B_{r}(T_{0})}{B_{r}(T_{0})(T-T_{0})}$$

(2) $$\beta =\frac{H_{cj}(T)-H_{cj}(T_{0})}{H_{cj}(T_{0})(T-T_{0})}$$

**Figure 2:**
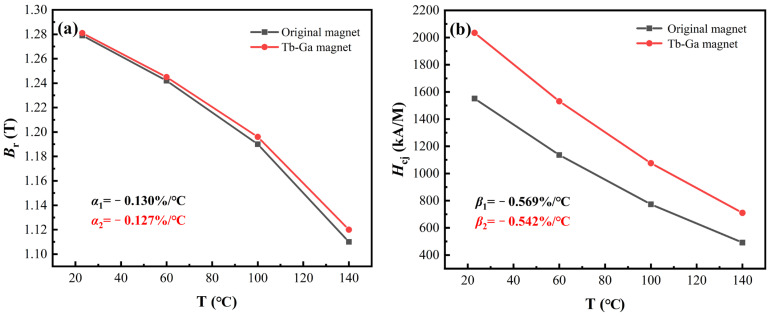
The temperature dependence for the B_r_ (**a**) and H_cj_ (**b**) of the original magnet and Tb-Ga magnet at the temperature range of 20–140 °C.

B_r_ (*T*_0_) and B_r_ (*T*) represent the remanence of the samples at room temperature (*T*_0_) and high temperature (*T*), respectively. H_cj_ (*T*_0_) and H_cj_ (*T*) refer to the coercivity of the samples at room temperature (*T*_0_) and high temperature (*T*). Figure 2a presents the remanence and B_r_ of the original magnet and the “Tb-Ga magnet” over the temperature range of 20 °C to 140 °C. Between 20 °C and 140 °C, the *α* value for the original magnet is −0.130%/°C, and for the “Tb-Ga magnet”, it is −0.127%/°C, indicating that the addition of the Tb and Ga composite diffusion source improves the temperature stability of the remanence. Moreover, Figure 2a shows that the B_r_ of the diffused magnets undergoes only a slight reduction across various temperature conditions, suggesting that the co-diffusion of Tb and Ga has a minimal effect on the magnet’s remanence. In comparison to the original magnet, the *β* value for the “Tb-Ga magnet” improves from −0.569%/°C to −0.542%/°C (as shown in Figure 2b), demonstrating a greater reduction in the absolute value of *β*. This is beneficial for maintaining better magnetic properties at higher temperatures. The coercivity temperature coefficient (*β*) is not only associated with the magnetic anisotropy field (*H*_A_) of the main phase grains but also with the internal microstructure of the magnet. In the “Tb-Ga magnet”, due to the diffusion of Tb elements, a (Tb, Nd)_2_Fe_14_B shell structure with a larger *H*_A_ forms around the NdFeB grains (as supported by the microstructural characterization discussed later in Section 2), which aids in reducing the *β* absolute value of the magnet. Additionally, the Ga element, with its lower melting point, may assist in optimizing the magnet’s microstructure, which also contributes to the reduction in the *β* absolute value. Consequently, *β* is effectively diminished. As a result, both the H_cj_ and thermal stability of the “Tb-Ga magnet” are improved at elevated temperatures.

**Figure 3:**
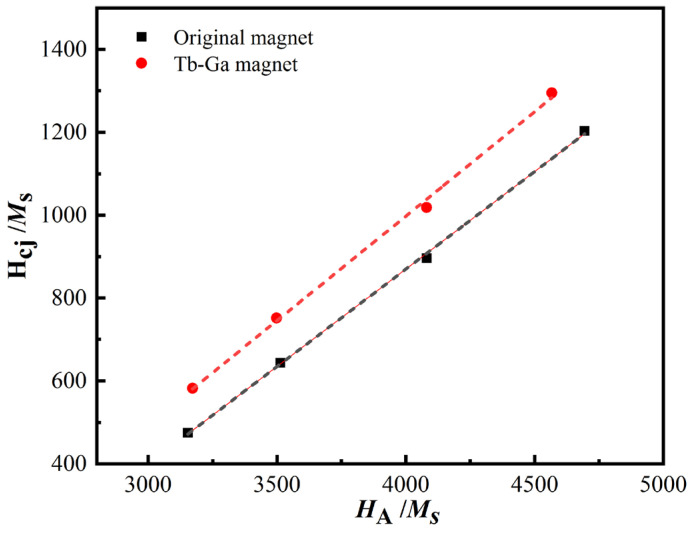
The dependence of H_cj_/M_s_ vs. *H*_A_/M_s_ of the original magnet and Tb-Ga magnet.

To examine the coercivity mechanism of magnets before and after diffusion, the nucleation field model [35,36] can be applied. The H_cj_ is described by the following equation:

(3) $$H_{cj}(T)=aH_{A}(T)-N_{eff}M_{S}(T)$$

where *H*_A_(*T*) represents the anisotropy field, *M*_s_(*T*) is the saturation magnetization, parameter *a* reflects the reduction in the anisotropy field due to microstructural defects or *c*-axis misalignment, and *N*_eff_ represents the microstructural factors that influence the local effective demagnetization field, which are mainly affected by grain size, grain shape, the presence of RE-rich triple-junction phases, and intergranular exchange [37,38]. By performing a linear fit of H_cj_/*M*_s_ versus *H*_A_/*M*_s_ at different temperatures, the parameters *a* and *N*_eff_ for the original magnet and “Tb-Ga magnet” are determined, as shown in Figure 3. From the results, it can be observed that the sensitive factor *a* increased from 4.70 to 5.04, indicating that the interfacial defects between the main phase and the Nd-rich phase in the magnet were optimized after diffusion. Simultaneously, the demagnetization factor *N*_eff_ increased from 1011.59 to 1018.53, suggesting that the stray field within the magnet increased post-diffusion, which may be related to grain growth during the diffusion process. Additionally, the incorporation of Tb-Ga may lead to misalignment of grains near the additive. These factors likely contribute to the increase in *N*_eff_, which significantly raises the local demagnetizing field [39].

**Figure 4:**
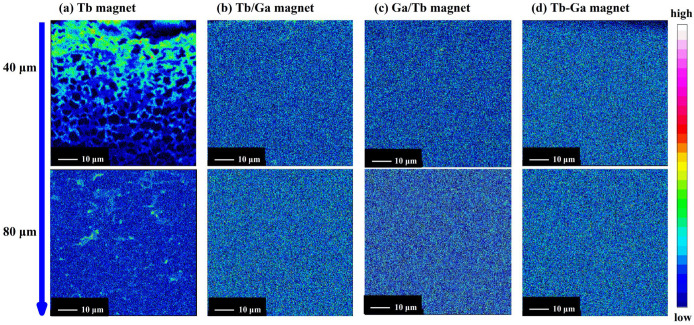
EPMA mappings of Tb in the 0–80 μm diffusion region for the Tb magnet (**a**), Tb/Ga magnet (**b**), Ga/Tb magnet (**c**), and Tb-Ga magnet (**d**).

Considering the increased intrinsic coercivity and improved thermal stability, it can be concluded that the Tb substitution efficiency in the “Tb-Ga magnet” is much better than in the other three diffusion magnets. The efficiency of Tb substitution in Nd-Fe-B sintered magnets is well known to be closely related to the distribution of the Tb element. To further explore the mechanism by which Ga enhances the efficiency of Tb substitution in sintered Nd-Fe-B magnets, the distribution of Tb from the diffusion interface to a depth of 80 μm was examined using EPMA, as shown in Figure 4. The results clearly show that the combined diffusion of Tb and Ga significantly reduces the accumulation of Tb at the diffusion surface of the magnets.

Analysis of Figure 4 shows that in the “Tb magnet”, a significant concentration of Tb atoms accumulates within a 20 μm region. Between 20 μm and 40 μm, the Tb atoms are distributed in a network-like pattern, and beyond 40 μm, the Tb content is very low. In contrast, the distribution of Tb atoms is much more uniform in the “Tb/Ga magnet”, “Ga/Tb magnet”, and “Tb-Ga magnet”. At a diffusion depth of 80 μm, all three mixed diffusion magnets show relatively high and more even concentrations of Tb, indicating that Tb atoms are able to spread into GB phases and diffuse into the hard magnetic matrix phases. The absence of effective diffusion channels in the “Tb magnet” results in significant lattice diffusion of Tb in the shallow surface layers of the diffusion process. As a result, a larger quantity of Tb is unable to diffuse deeper into the layers along the grain boundaries, thereby reducing the efficiency of Tb diffusion. In contrast, the addition of low-melting-point elements in the “Tb/Ga magnet”, “Ga/Tb magnet”, and “Tb-Ga magnet” enhances the wettability of the Nd-rich grain boundary phases, and increases the efficiency of Tb diffusion along the grain boundaries.

**Figure 5:**
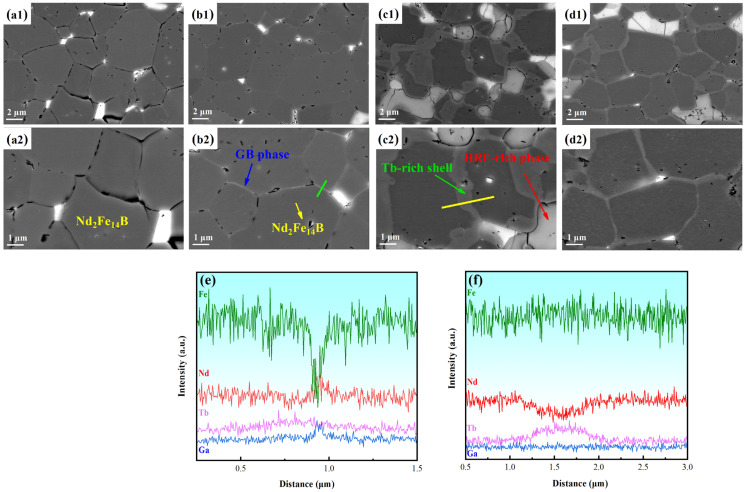
Cross-sectional BSE-SEM images of the Tb magnet (**a1,a2**), Tb/Ga magnet (**b1,b2**), Ga/Tb magnet (**c1,c2**), and Tb-Ga magnet (**d1,d2**). (**e**) Concentration profiles of line scanning in (**b**). (**f**) Concentration profiles of line scanning in (**c**).

Figure 5 shows the BSE-SEM images of the magnets at a diffusion depth of 100 μm from the diffusion surface, with the corresponding line scans provided in Figure 5e,f. As depicted in Figure 5(a1,a2), in the “Tb magnet”, a large portion of the Nd_2_Fe_14_B main phase grains (black, indicated by yellow arrow) are in direct contact. As shown in Figure 4a, the Tb concentration sharply decreases beyond 40 μm, making the formation of a core–shell structure difficult. This direct contact between the matrix grains results in exchange coupling among the ferromagnetic grains, which reduces the intrinsic coercivity of the sample.

The “Tb/Ga magnet” shows a predominantly smooth, thin, flaky grain boundary (GB) phase (white, indicated by blue arrow). Figure 5e indicates that the continuous GB phase is rich in Nd and Ga but lacks Fe. This supports the idea that the low-melting-point Ga element aids in forming thin grain boundaries. Ga’s low melting point further reduces the melting point of the GB phase, improving its fluidity and wettability. This promotes the formation of thin grain boundary phases that surround the Nd_2_Fe_14_B main phase, reducing the exchange coupling between adjacent ferromagnetic grains. As a result, the intrinsic coercivity (H_cj_) of the magnets improves [40,41]. However, in the “Tb/Ga magnet”, the core–shell structure is also not as distinct, though EDS results show that Tb slightly increases between the grain boundaries and grains. This is because, as mentioned earlier, a significant amount of Tb is lost during the Ga film deposition process, leading to a reduction in the Tb diffusion source. Consequently, only a small amount of Tb enters the main phase grains, which is insufficient to form a clear core–shell structure at the 100 μm depth observed.

In comparison to the “Tb magnet”, the “Ga/Tb magnet” shows a thicker Tb-rich shell structure (gray, indicated by green arrow). Figure 5f illustrates that the shell structure is rich in Tb but lacks Nd, indicating that a considerable amount of Tb has infiltrated into the main phase grains, leading to the formation of the (Tb, Nd)_2_Fe_14_B phase. This is attributed to the low melting point element Ga, which enhances the fluidity and wettability of the GB phase, thereby creating more effective diffusion pathways. Since Tb mainly diffuses along the GB phase, the addition of Ga significantly promotes the movement of Tb into deeper parts of the magnet, causing a higher accumulation of Tb around the main phase. Consequently, Tb easily diffuses into the main phase, forming the Tb_2_Fe_14_B phase, and resulting in the development of a thicker shell structure. This increases the nucleation field of reverse magnetic domains at the edge of main-phase grains, thereby enhancing coercivity [42,43]. However, it is known that overly thick Tb-rich shells can reduce the efficiency of Tb utilization [1]. Given the fixed amount of the Tb diffusion source, the formation of a thick shell reduces the amount of Tb that can penetrate deeper into the magnet. Moreover, many HRE-rich phases appear in bright gray (indicated by the red arrow), suggesting that a large portion of the Tb has replaced Nd atoms in the Nd-rich phase rather than entering the main phase to form Tb_2_Fe_14_B, leading to further Tb waste.

In the “Tb-Ga magnet”, a thinner and more uniform shell–core structure is readily observable. Unlike the layer-by-layer deposition of Tb and Ga, the simultaneous deposition of both elements allows Tb to diffuse deeper into the magnet instead of concentrating at the main phase grains, resulting in a thinner shell structure, which contributes to the magnet’s improved magnetic properties. The deeper diffusion of Tb leads to the formation of more shell–core structures. In conclusion, the structure of the “Tb-Ga magnet” positively influences coercivity and exhibits the best overall magnetic properties among the four diffusion magnets.

**Figure 6:**
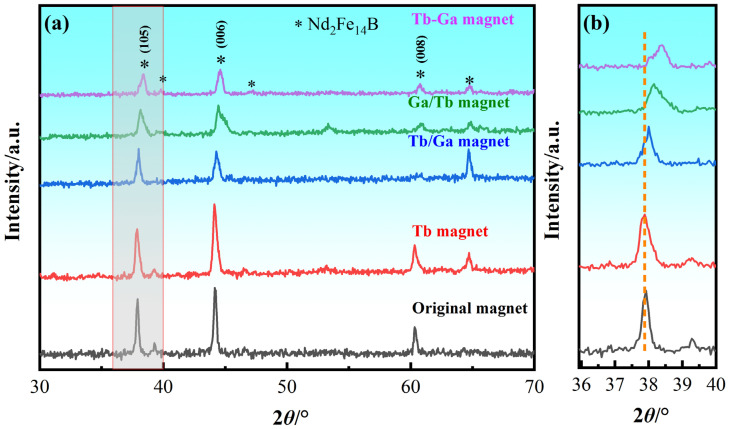
(**a**) XRD patterns of the different magnets. (**b**) The enlarged patterns in the range of 36~40°.

Figure 6 shows the XRD patterns of the original magnet, the “Tb magnet”, “Tb/Ga magnet”, “Ga/Tb magnet”, and “Tb-Ga magnet”. All XRD patterns exhibit peaks corresponding to Nd_2_Fe_14_B [39,44]. When compared to the original magnet, the main phase (006) peak of the diffused magnets shifts towards higher angles. According to the Bragg equation, this shift in peak angle corresponds to a reduction in the crystal lattice parameters. Since the radius of Tb^3+^ is smaller than that of Nd^3+^, the matrix phase unit cell contracts as Tb atoms diffuse into it, causing the peak to shift to higher angles. The more pronounced the shift, the greater the amount of Tb diffused into the main phase Nd_2_Fe_14_B. As shown in the figure, the “Ga/Tb magnet” and “Tb-Ga magnet” exhibit higher Tb contents, substituting Nd in the main phase compared to the other diffused magnets, which results in the formation of more (Tb, Nd)_2_Fe_14_B phases. Furthermore, the orientation degree of sintered Nd_2_Fe_14_B magnets can be estimated by the intensity ratio of (00l)/(105) peaks. The intensity ratios for the three mixed diffusion magnets are lower than those of the original magnet and the “Tb magnet”, suggesting that the c-axis crystallographic texture of the magnet has been weakened.

**Figure 7:**
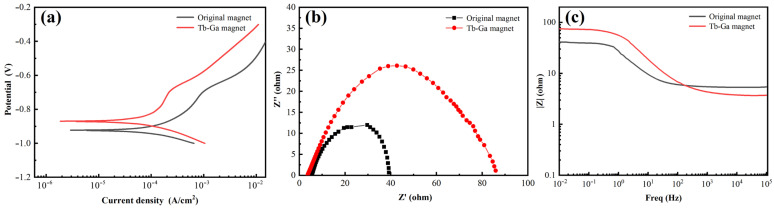
(**a**) Potentiodynamic polarization curves of the original magnet and Tb-Ga magnet, (**b**) Nyquist and (**c**) Bode plots of the original magnet and Tb-Ga magnet in a 3.5 wt.% NaCl aqueous solution.

In this experiment, the original magnet and the Tb-Ga magnet were immersed in a 3.5 wt.% NaCl aqueous solution for 1 h to allow the open circuit potential on the surface to stabilize. Following this, the potentiodynamic polarization curves for both samples were recorded. The polarization curves shown in Figure 7a reveal that, in comparison to the original magnet, the curve for the Tb-Ga magnet shifts upward and to the left. This indicates that the Tb-Ga magnet has a more positive corrosion voltage (*E_corr_*) and a lower corrosion current density (*I_corr_*). The fitting parameters for the polarization curves, presented in Table 1, clearly indicate that the Tb-Ga magnet exhibits better corrosion resistance than the original magnet. The corrosion voltage (*E_corr_*) for the Tb-Ga magnet slightly increased from −0.923 V to −0.869 V, while the corrosion current density (*I_corr_*) grew by an order of magnitude, from 2.87 × 10^−6^ A∙cm^−2^ to 1.89 × 10^−6^ A∙cm^−2^. The Nyquist and Bode plots for the original magnet and the Tb-Ga magnet are presented in Figure 7b,c. As illustrated in Figure 7b,c, and Table 1, compared to the original magnet, the polarization resistance (Rp) of the Tb-Ga magnet increased from 4.92 Ω∙cm^−2^ to 32.09 Ω∙cm^−2^, and the impedance modulus values (|Z|_0.01Hz_) showed a significant increase, further confirming the positive effect of adding Tb-Ga to the NdFeB magnet [45,46].

Overall, the electrophoretic deposition of Tb-Ga not only improves the magnet’s magnetic properties but also enhances its corrosion resistance, making it a coating with excellent overall performance.

## 3. Materials and Methods

Commercial sintered Nd-Fe-B magnets were selected as the starting material for the EPD-based GBDP study. The original magnet has an H_cj_ of 1527.87 kA/m, a (*BH*)*_max_* of 318.31 kJ/m^3^, and a remanence (B_r_) of 1.28 T. The magnets were cut into thin-sheet samples with dimensions of (10 ± 0.1)mm × (10 ± 0.1)mm × (3 ± 0.1) mm (c-axis) using wire cutting. The samples were then polished with 800, 1000, and 2000 mesh sandpaper, followed by immersion in a 3 vol% HCl solution for 15 s. Afterward, they were ultrasonically cleaned with water, anhydrous ethanol, and then dried to prepare for electrophoretic deposition.

**Figure 8:**
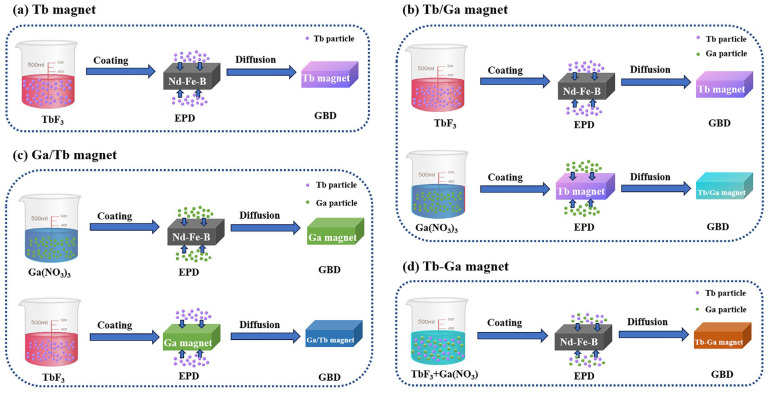
Schematic diagrams of GBD process of (**a**) Tb magnet, (**b**) Tb/Ga magnet, (**c**) Ga/Tb magnet and (**d**) Tb-Ga magnet.

For the EPD, TbF_3_ powder with an average particle size of 0.3 μm and Ga(NO_3_)_3_ (Macklin, 99.9%) powder with an average size of 50 nm were used. The TbF_3_ and Ga(NO_3_)_3_ powders were mixed in an ethanol suspension. Platinum plates were employed as the anode, and the magnet was used as the cathode during the electrophoresis process. Four deposition processes were chosen to evaluate the impact of the electrophoretic deposition sequence on magnet performance. The deposition sequence and the corresponding deposited solutions are outlined in Table 2. To ensure uniformity across the experiments, the Tb concentration in the magnet was maintained at a mass fraction of 0.5 ± 0.01%. After deposition, the samples underwent heat treatment at 900 ± 5 °C for 7 h and at 500 ± 5 °C for 2 h under a vacuum of 6 ± 0.2 × 10^−4^ Pa to complete the GBDP. Figure 8 shows the schematic diagrams of the four GBDP diffusion processes.

In the following section, the magnets subjected to diffusion are referred to as the Tb magnet, Tb/Ga magnet, Ga/Tb magnet, and Tb-Ga magnet, based on the different sequences of electrophoretic deposition used.

The corrosion resistance of sintered Nd-Fe-B magnets, both before and after electrophoretic deposition and grain boundary diffusion, was tested using a CHI660D electrochemical workstation from Chenhua Instrument Co., Ltd, Shanghai, China. The experiment employed a conventional three-electrode setup, where the sample acted as the working electrode, a saturated calomel electrode was used as the reference electrode, and a platinum electrode served as the auxiliary electrode. The exposed area of the sample was 2.68 cm^2^, with a 3.5 wt.% NaCl solution as the corrosion medium. The samples were immersed for 1 h, with a scanning rate of 0.02 mV/s, and the tests were conducted at room temperature. Impedance spectroscopy was performed, scanning from a high frequency of 10^5^ Hz to a low frequency of 10 mHz, with an amplitude of 10 mV.

Magnetic properties of the magnets were measured using a pulsed field magnetometer (PFM Hirst). The morphology and elemental analysis of the samples were performed using scanning electron microscopy (SEM ZEISS Sigma 360) with a backscattered electron (BSE) detector and energy dispersive spectroscopy (EDS). The phase structures of the magnets were analyzed by X-ray diffraction (XRD XD-2). Additionally, an electron probe microanalyzer (EPMA JXA-8100) was utilized to study the elemental distribution within the samples.

## 4. Conclusions

To summarize, a Tb and Ga mixed diffusion source was prepared via the EPD technique. The magnetic properties, microstructure, and elemental distribution of the magnets were evaluated, leading to the following conclusions:

(1) The sequence of Tb and Ga deposition greatly affects the coercivity of the magnet. Among the deposition methods, the co-deposition of Tb and Ga resulted in the most significant enhancement in magnetic performance. The Tb substitution efficiency for Nd and the thermal stability of the Tb and Ga mixed diffusion magnets are notably superior to those without Ga.
(2) In comparison to layered deposition of the mixed diffusion sources, the simultaneous deposition method enhances the diffusion depth, optimizing the Tb distribution and improving the utilization of HRE resources by preventing the formation of overly thick Tb-rich grain boundaries.
(3) Electrochemical testing shows that the mixed diffusion magnets exhibit higher corrosion voltage (*E_corr_*), lower corrosion current density (*I_corr_*), and increased polarization resistance (*R*_p_), significantly improving the corrosion resistance of Nd-Fe-B magnets.

Continued research may facilitate the large-scale industrial production of magnets with both excellent magnetic properties and enhanced corrosion resistance. This advancement could be realized through the co-diffusion of low melting point elements with heavy rare earth metals in Nd-Fe-B sintered magnets.

## Figures and Tables

**Table 1:** The fitting parameters of potentiodynamic polarization curves.

Sample	*E_corr_* (V)	*I_corr_* (A∙cm^−2^)	*R*_p_ (Ω∙cm^−2^)
Original magnet	−0.923	2.87 × 10^−6^	14.92
Tb-Ga magnet	−0.869	1.89 × 10^−6^	32.09

**Table 2:** Electrophoretic deposition setup and process parameters.

Magnet Name	Deposition Setup	Deposited Solution	Deposition Parameters
Tb magnet	Only Tb coatings were deposited on the surface of the magnet.	10 g/L TbF_3_ ethanol suspension.	U = 120 ± 0.1 V; *t* = 1 min
Tb/Ga magnet	Tb and Ga coatings were deposited layer by layer on the surface of the magnet.	10 g/L TbF_3_ ethanol suspension;1.5 g/L Ga(NO_3_)_3_ ethanol suspension.	
Ga/Tb magnet	Ga and Tb coatings were deposited layer by layer on the surface of the magnet.	10 g/L TbF_3_ ethanol suspension;1.5 g/L Ga(NO_3_)_3_ ethanol suspension.	
Tb-Ga magnet	Both Tb and Ga coatings were deposited simultaneously on the surface of the magnet.	the 10 g/L TbF_3_ and 1.5 g/L Ga(NO_3_)_3_ mixed ethanol suspension.	

## Data Availability

Data will be made available on request.

## Abbreviations

The following abbreviations are used in this manuscript:

HRE	Heavy rare earths
GBDP	Grain boundary diffusion process
EPD	Electrophoretic deposition
PFM	Pulsed field magnetometer
SEM	Scanning electron microscopy
BSE	Backscattered electron
EDS	Energy dispersive spectroscopy
EPMA	Electron probe microanalyzer
XRD	X-ray diffraction
